# Dynamic visual cortical connectivity analysis based on functional magnetic resonance imaging

**DOI:** 10.1002/brb3.1698

**Published:** 2020-06-07

**Authors:** Le Zhao, Weiming Zeng, Yuhu Shi, Weifang Nie, Jiajun Yang

**Affiliations:** ^1^ Laboratory of Digital Image and Intelligent Computation Shanghai Maritime University Shanghai China; ^2^ Department of Neurology Shanghai Sixth People's Hospital East Affiliated to Shanghai University of Medicine & Health Science Shanghai China

**Keywords:** dynamic effective connectivity, dynamic functional connectivity, fMRI, Granger causality, visual cortex

## Abstract

**Background:**

Studies of brain functional connectivity (FC) and effective connectivity (EC) using the functional magnetic resonance imaging (fMRI) have advanced our understanding of functional organization on visual cortex of human brain. The current studies mainly focus on static or dynamic connectivity, while the relationships between them have not been well characterized especially for static EC (sEC) and dynamic EC (dEC), as well as the consistency characteristics of changing trend of dFCs and dECs, which is of great importance to reveal the neural information processing mechanism in visual cortex region.

**Method:**

In this study, we explore these relationships among several subareas of human visual cortex (V1–V5) by calculating the connection intensity and information flow among them over time by sliding window method, which are defined by Pearson correlation coefficient and Granger causality analysis, respectively, in each window.

**Results:**

The results demonstrate that there are extensive connections existing in human visual network, which are time‐varying both in resting and task‐related states. sFC intensity is negatively correlated with the variance of dFC, while sEC intensity is positively correlated with the variance of dEC. Furthermore, we also find that dFC within visual cortex at rest shows more consistency, while dEC shows less compared with task state in changing trend.

**Conclusion:**

Therefore, this study provides novel findings about dynamics of connectivity in human visual cortex from the perspective of functional and effective connectivity.

## Graphical abstract

We explore relationships among several subareas of human visual cortex (V1–V5) by calculating the connection intensity and information flow among them over time by sliding window method, which are defined by the Pearson correlation coefficient and Granger causality analysis, respectively, in each window. The study provides novel findings about dynamics of connectivity in human visual cortex from the perspective of functional and effective connectivity.

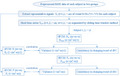

## 1 INTRODUCTION

Functional magnetic resonance imaging (fMRI) mainly refers to blood oxygen level‐dependent fMRI (BOLD‐fMRI), which has the advantages of noninvasive, repeatable, and high spatial resolution, and has been applied to various aspects of clinical and basic research (Logothetis, Pauls, Augath, Trinath, & Oeltermann, 2001). Over the past two decades, the study of functional specificity and functional integration has led to the development of fMRI. The functional specificity study only focused on the location of important brain functions and the functional activities of local brain regions, while ignoring the interrelationships between different brain regions and providing only a small part of the brain structure and function (Glasser et al., 2016). Functional integration is described in terms of functional connectivity (FC) and effective connectivity (EC) (Friston, Frith, & Frackowiak, 1993). The functional connectivity describes the temporal correlations between spatially remote neurophysiological events. There are two kinds of research methods: One is hypothesis‐driven method, which mainly includes correlation analysis (Tian et al., 2010; Zhang et al., 2013), coherent analysis, and generalized linear model (GLM); the other is data‐driven method, which mainly includes independent component analysis (ICA) (Shi, Zeng, Wang, & Chen, 2015; Shi, Zeng, Wang, & Zhao, 2018), principal component analysis (PCA), and cluster analysis methods. The EC reflects the directional connectivity between different neural units or brain regions and forms a network with edges representing directed weights of one neuron or brain region relative to the other. The models for studying the brain's effective connectivity include structural equation model (SEM) (Bavelier et al., 2000), transfer entropy (Vicente, Wibral, Lindner, & Pipa, 2011), dynamic causal model (DCM) (Xin & Biswal, 2014), and Granger causality analysis. Among these, Granger causality method is a statistical method for investigating the flow of information between time series, which does not require prior knowledge and emphasizes the trait of time sequence when analyzing data interactions. So, it has been widely applied by neuroscientists to diverse sources of data, including electroencephalography (EEG), magnetoencephalography (MEG), fMRI, and local field potentials (LFP) (Dimitriadis, Laskaris, Tsirka, Vourkas, & Micheloyannis, 2012; Gao et al., 2015).

BOLD‐fMRI studies have traditionally investigated patterns of FC and EC that are static within the scanning period. However, studies in recent years have shown that the connectivity of the brain regions has instantaneous changes, and the dynamics of this connectivity are reflected in the brains during a task or at rest (Bassett et al., 2011; Hutchison, Womelsdorf, Gati, Everling, & Menon, 2013). Studying the time‐dependent information of the brain connectivity helps humans to have a more comprehensive understanding of the brain's functional and structural organization, so dFC and dEC analyses have become a new exploration field in brain connectivity research though the dynamic changes have hitherto been overlooked in fMRI studies most likely due to the poor temporal resolution of fMRI especially in dEC. The common sliding window method uses a moving window to divide the entire BOLD signal into multiple short signals (Tobia, Hayashi, Ballard, Gotlib, & Waugh, 2017). Different windows can obtain multiple functional connectivity and effective connectivity matrices to reflect the dynamic brain network connectivity. Dynamic FC often occurs within the same individual and is clearly relevant to behavior. Some researchers believe that it may be heavily related to high‐level thought or consciousness (Hutchison, Womelsdorf, Allen, et al., 2013). It is also associated with a variety of different neurological disorders and can potentially serve as disease biomarkers (Kaiser et al., 2016). Previous studies have also found that the effective connectivity exhibits changes across cortex of human brain (Hu, Zhang, & Hu, 2012; Spadone, et al., 2015). Compared with sFC and sEC based on the traditional fMRI time series analytical methods, dynamic connectivity technology can better reflect the dynamic participation of different brain regions in the actual brain, which has been suggested to be a more accurate representation of functional brain networks.

Functional magnetic resonance imaging has made some progress in the basic research of normal human brain functional networks (visual, auditory, motor, sensory, etc.). The study of visual cortex is the earliest field of application of fMRI, which is mainly relevant to the easy control of visual stimulation conditions, and the relatively large intensity of the visual cortex activation signal. In visual research, when a subject receives a certain kind of visual stimuli, the visual signal is transmitted through the visual pathway to the visual cortex, and the increase of neuronal activity for processing relevant visual information causes local blood flow to change. The fMRI can reflect the location, range, and intensity of neuron activity and has become an effective method for visual research. The first human brain fMRI obtained by Belliveau et al. (1991) in 1991 was related to visual research and created a historical precedent for the study of fMRI in the localization of human brain function. The results showed a significant increase in the volume of blood flow in the primary visual cortex after visual stimulation, and the extent and coordinates of brain activation were reported. Research on the anatomy and physiology of the visual cortex of primates has provided valuable information for the study of the human visual cortex. Through these studies, it has been found that the human visual cortex is homologous to the visual cortex of primates and confirmed that humans have at least 25 visual cortical areas, which cover more than half of the cortical area (Sereno et al., 1995). In recent years, the BOLD‐fMRI method has been used to located accurate visual subregions such as V1, V2, V3, V4, and MT/V5, which is basically consistent with the traditional view (Warnking et al., 2002).

In this study, we adopted fMRI data considering research on sFC, sEC, dFC, and dEC in both task‐related and resting states. As compared with literature of dynamic FC and EC, the novelty of this study is threefold. First, most of previous studies were focused on difference of (a) FC or EC between tasks and rest to observe the modulation effect of tasks on brain network connectivity (Spadone, et al., 2015), (b) FC between task and control periods during a block design experiment (Di et al., 2015), or (c) dynamic changes in FC during tasks or at rest (Allen et al., 2014; Gonzalez‐Castillo & Bandettini, 2017). However, our study is aimed to investigate changes in FC and especially EC at the same time over time in normal subjects at rest and during a task with repeatedly presented identical stimuli, which may provide new information on the dynamic recombination of cerebral cortex under visual stimulation. Second, the relationship between intensity of sFC and variance of dFC (Fong et al., 2019) has gained attention in recent years but not in EC, so it is going to be discussed in this paper. Third, the dynamics of functional connectivity is usually characterized by its own variance, which is viewed within a partial perspective and is clearly not enough. The dFC or dEC between two certain brain regions can be viewed as a vector, which is described as the changing trend with elements calculated in all windows. Therefore, we studied the consistency of changing trend, which reflects the covariation relationship of dFCs or dECs on the whole. In other words, FCs or ECs describe the undirected or directed relationship among time series of brain regions obtained from fMRI scans, while the consistency of changing trend describes the relationship between time‐varying FCs which no longer describes a single dFC. We explore it in the present study to further investigate the dynamic characteristics of brain connectivity. The dynamics study of FC and EC in this paper is divided into three steps: (a) A sliding window method was used to estimate the time‐varying correlation coefficient and Granger causality among V1–V5 of visual subregions (Luo et al., 2016); (b) the relationship between the intensity of static FC and variance of dynamic FC, and the intensity of static EC and variance of dynamic EC was calculated, respectively; (c) the consistency of changing trend in dFC and dEC was estimated to validate the connectivity dynamics from a global perspective. The results showed that there were indeed extensive connections between various brain regions of the visual system, and the network of brain regions was dynamic both in rest and task states. Static functional connection intensity is negatively correlated with the variance of dynamic FC, while static effective connection intensity is positively correlated with the variance of dynamic EC. We can also find that dFC within visual cortex at rest shows more consistency, while dEC shows less compared with task state. In conclusion, dynamic brain connectivity analysis is expected to be a more accurate representation of functional brain networks and may shed a bright light on a variety of vision‐related disorders.

## 2 MATERIALS AND METHODS

### 2.1 Participants and fMRI data acquisition

Resting‐state and task‐related fMRI data were collected from the enhanced Nathan Kline Institute (NKI)/Rockland sample of the international neuroimaging data‐sharing initiative (INDI) (http://fcon_1000.projects.nitrc.org/indi/enhanced/) (Nooner, Colcombe, Tobe, Mennes, & Milham, 2012). Institutional Review Board Approval was obtained for this project at the Nathan Kline Institute and at Montclair State University. Written informed consent was obtained for all study participants. Only the resting‐state and block‐designed visual checkerboard data with a relatively short repetition time (TR) of 645 ms were used in the current analysis, which could provide necessary high temporal resolution to unravel FC and EC dynamics. In total, 53 subjects (18–41 years, mean = 23.3 years, standard deviation = 5.6 years) in session DS2 from this dataset were included in the current study.

**FIGURE 1:**
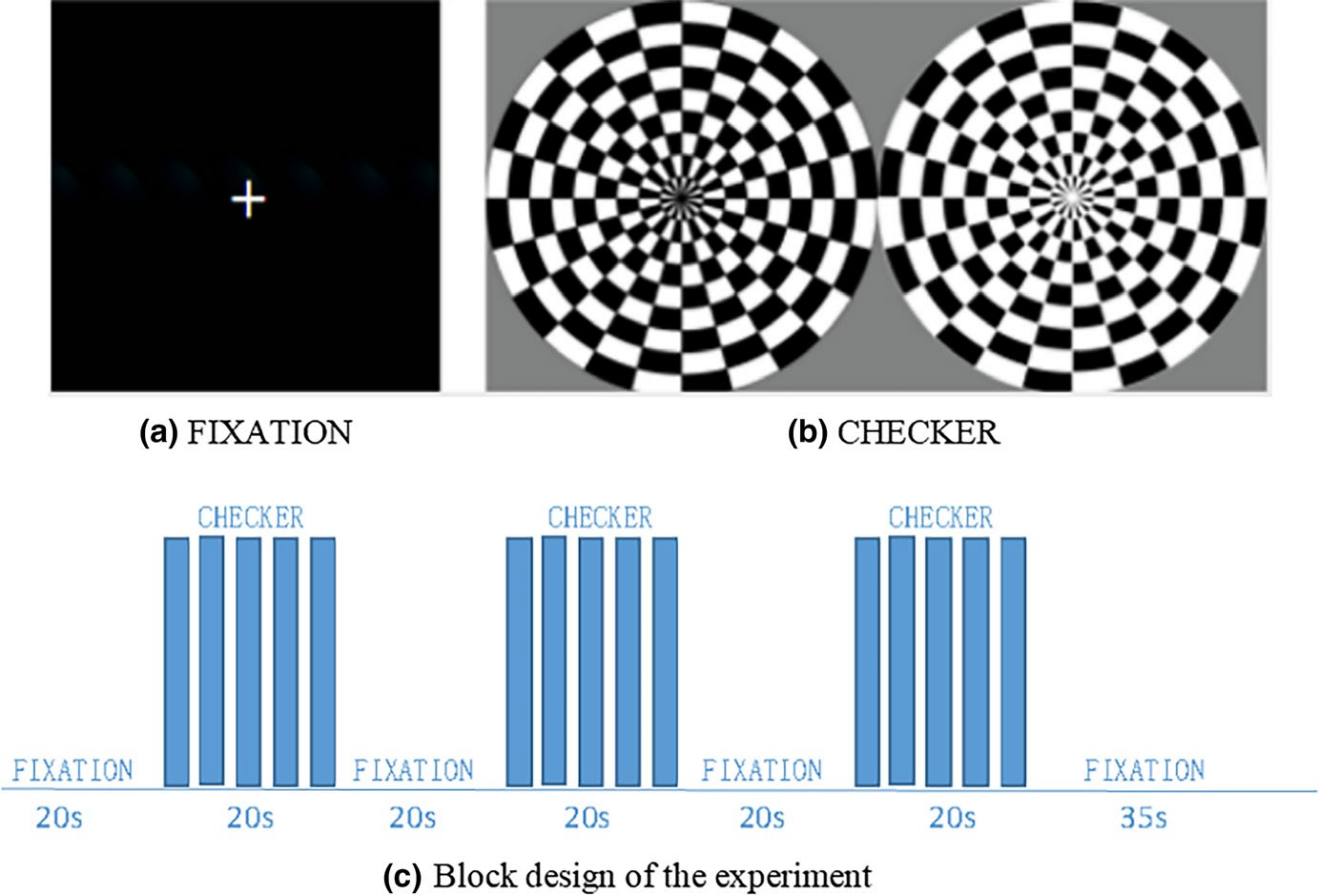
Verification test of visual stimulation. (a) A black screen during fixation state, (b) picture of checkerboard patterns after stain. (c) Block design of task‐related fMRI experiment. The scan started with a 20‐s fixation condition and followed by a 20‐s checkerboard condition with three repetitions. After the third checkerboard block, there was an additional 35‐s fixation condition

The task‐related fMRI data were recorded from a simple checkerboard visual experiment, where the checkerboard stimuli were presented in the center of the screen with a flickering frequency of 4 Hz. There was a black‐and‐white flipped checkerboard with radial shape during the stimulus state, and a cross on the black screen during the control state is shown in Figure 1. The block types are [FIXATION, CHECKER, FIXATION, CHECKER, FIXATION, CHECKER, FIXATION] (see Figure 1), with seven blocks in all. The total scan time was about 2 min 35 s with totally 240 images acquired. The resting‐state and task‐related fMRI data were all scanned using a multiband echo‐planar imaging (EPI) sequence with the following parameters: TR/TE = 645/30 ms; acquisition matrix = 74 × 74; flip angle = 60°; voxel size = 3 mm^3^ isotropic; slices = 40.

### 2.2 Data preprocessing

Data were preprocessed using an automated pipeline based around DPARSF (Yan & Zang, 2010) software package. Preprocessing included the removal of the first 10 image volumes, motion correction, spatial normalization into Montreal Neurological Institute space, reslicing to 3 mm × 3 mm×3 mm voxels, and smoothing with a Gaussian kernel (FWHM = 4 mm), detrending and nuisance covariates regression (six parameters related to head movement, white matter, and CSF signals). Poor‐quality scans with nonstationary and excessive head motion, defined a priori as >2 mm translation, or >2° rotation, were excluded from analysis; this included six resting runs and two task runs, so 45 subjects were included in the final analysis.

### 2.3 ROI selection

**FIGURE 2:**
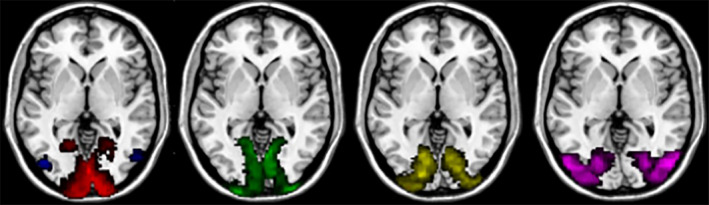
Selection of V1–V5 as ROIs. Five ROIs used in the current analyses are displayed in red (V1), green (V2), yellow (V3), violet (V4), and blue (V5) according to PMaps of SPM anatomy toolbox

The probability map in the SPM anatomy toolbox (Eickhoff et al., 2005) is used to select V1–V5 as the ROIs, which are shown in Figure 2. The mean time series for regions of interest (ROI) was extracted for each subject of resting and task‐related fMRI data with REST 1.8 software (http://restfmri.net/forum/REST_V1.8).

### 2.4 Method

#### 2.4.1 Static functional connectivity (sFC)

The static functional connection matrix *R* (size: *m* × *m*) is computed as the Pearson correlation coefficient matrix between the average time series of ROIs *X_t_* (*i* = 1,2,…,*m*) (*m* is the number of ROIs) over the entire scan time with *R_ij_* = *R_ji_* = corr(*X_i_*,*X_j_*) and then averaged across all subjects in each group, respectively. To avoid repeated information, only the lower triangular portion of the symmetrical FC matrix was properly converted into a static FC intensity vector *R_s_* (size: $1\times \frac{m^{2}-m}{2}$ ) for further analysis. In this study, there are five ROIs and each subject has ten functional connectivity strength values.

#### 2.4.2 Static effective connectivity (sEC)


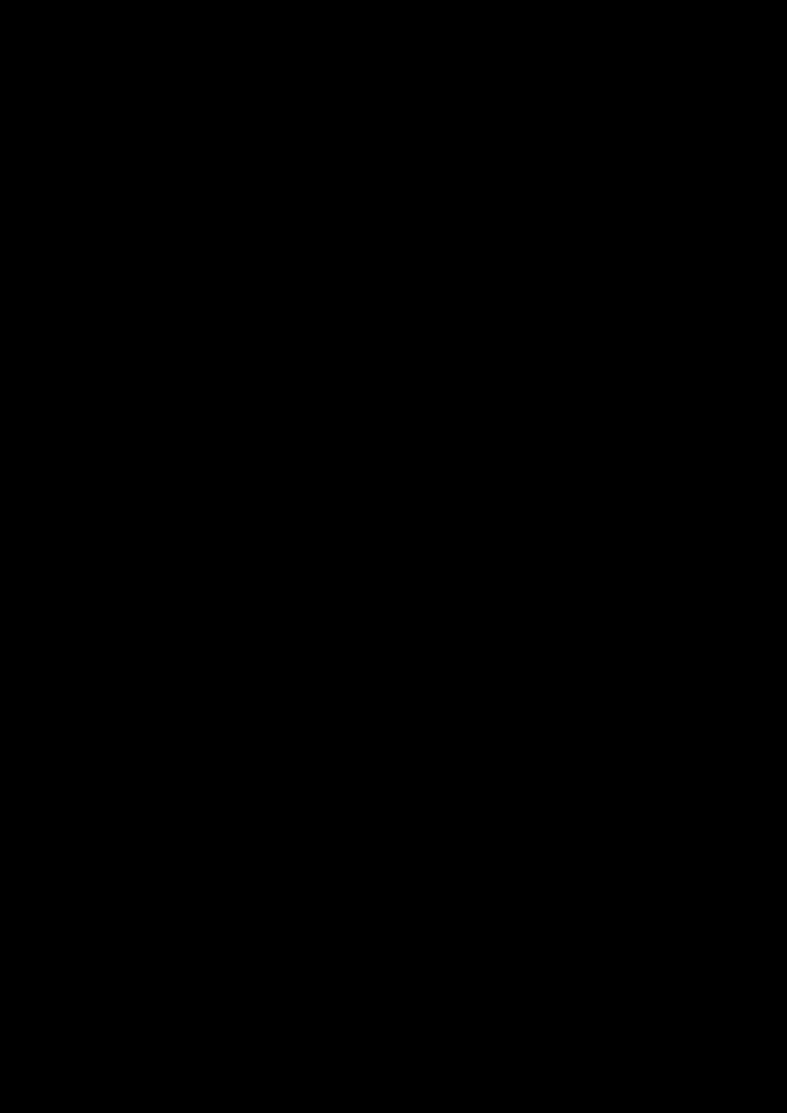
 Granger causality analysis (GCA) method is used in this article, which refers to a predictive relationship among time series. Generally speaking, given two time series *X*(*n*) and *Y*(*n*) (*n* = 1,2,…,*t*), we say that YG causes X if it would be more favorable in predicting X with the incorporation of Y's historical information than only using X's historical information. In order to check whether YG causes X conditional on Z (given Z), the vector autoregressive (VAR(*p*)) and joint autoregressive model are described as:

(1) $$X_{t}=\sum_{i=1}^{p}a_{1i}X_{t-i}+\sum_{i=1}^{p}c_{1i}Z_{t-i}+\xi_{1t}$$

(2) $$X_{t}=\sum_{i=1}^{p}a_{2i}X_{t-i}+\sum_{i=1}^{p}c_{2i}Z_{t-i}+\sum_{i=1}^{p}b_{2i}Y_{t-i}+\xi_{2t}$$

where *a*_1_ *_i_*,*c*_1_ *_i_*,*a*_2_ *_i_*,*c*_2_ *_i_* and *b*_2_ *_i_* are best regression parameters of the model, $\xi_{1t}$ and $\xi_{2t}$ are two zero‐mean uncorrelated white‐noise series. The model order *p* can be determined by BIC criterion. $\text{var}(\xi_{1t})$ and $\text{var}(\xi_{2t})$ represent the estimation accuracy of the X's current value with the past behavior of X and the past behavior of X joint with Y in condition of Z, respectively. The measure of the strength of the causality Y → X in condition of Z can be defined as,

(3) $$F_{Y\to X|Z}=\ln\frac{\text{var}(\xi_{1t})}{\text{var}(\xi_{2t})}$$

If there is no direct causality between Y and X but an indirect causal relationship between them because of Z, *b*_2_ *_i_* = 0 in (2) and $\text{var}\left(\xi_{1t}\right)=\text{var}\left(\xi_{2t}\right)$ , resulting in $F_{Y\to X|Z}=0$ . It means that under the condition of Z, adding Y to the model does not improve the prediction accuracy.

We use the code provided in Luca Faes's paper (Faes, Nollo, Stramaglia, & Marinazzo, 2017) to calculate the effective connectivity between ROIs and obtain the static effective connection matrix *F* (size: *m* × *m*) for all subjects, which were then averaged in each group, respectively, with the model order *p* optimized separately for each subject using the BIC criterion. The static EC intensity vector *F_s_* (size: $1\times (m^{2}-m)$ ) is defined as the effective connectivity strength between ROIs during the entire scan time period, that is, we removed the diagonal from *F* and then converted it into a row vector. In this study, there are five ROIs and each subject has twenty effective connectivity strength values.

#### 2.4.3 dFC and dEC

Static connectivity methods assume networks in the brain are stationary over the whole scan length (typically ranging from 6–10 min), which represents an average state. However, dynamic connectivity methods regard the networks as a function of time with variability often quantified as ALFF‐FC map (Allen et al., 2014; Qin, Chen, Hu, Zeng, & Shen, 2015), the index of dispersion (variance/mean) (Demirtaş et al., 2016; Tian, Li, Wang, & Yu, 2018), or simple variance (Fong et al., 2019; Jin et al., 2017) of the dFC, which is like higher order statistics of connectivity. However, previous studies often aim at static and dynamic FCs. The dynamic property of the EC especially the relationship between static EC and dynamic EC is so far overlooked.

Based on the sliding window method, dynamic functional and effective connectivity network for each subject were calculated using the defined V1–V5 as the ROIs. The BOLD signal $X_{i}\;\left(i=1,2,...,m\right)$ of the ROI is segmented into a short time series $X_{i,w}\;\left(i=1,2,...,m;w=1,2,...,n\right)$ . m is the number of ROIs and n is the number of windows. The window width often ranged from 8 to 240 s (Shakil, Lee, & Keilholz, 2016) in the study of dynamic brain network connectivity previously. Granger pointed out that sample size is an important factor influencing causality. Zhou and Zinai (2004) tested two stationary sequences with the first‐order lag model and found that the probability of Granger causality increased significantly with the increase of sample size. In this paper, the number of time points for each window is set to 100 and the step size is set to 1, so *n* = 131 for fMRI data. The functional connection matrix $R_{w}$ (size: *m* × *m*) corresponding to the window is calculated by $X_{i,w}\;\left(i=1,2,...,m;w=1,2,...,n\right)$ , see formula (4), where the element value of the i‐th row and the j‐th column is indicated as $R_{w}\left(i,j\right)$ , and corr represents the calculation of the Pearson correlation coefficient. Therefore, $R_{w}\left(w=1,2,...,n\right)$ obtained by each subject can reflect the dynamic brain functional connectivity network of the subject. The functional connection matrix of all subjects was averaged to obtain the dynamic functional connectivity matrix (dFCM, size: *m* × *m* × *n*) for each group. At the same time, the effective connection matrix $F_{w}$ (size: *m* × *m*) corresponding to each window is calculated by Granger causality analysis method, with $F_{w}\left(i,j\right)$ representing the effective connection value from the *j*‐th ROI to the *i*‐th ROI. $F_{w}\left(w=1,2,...,n\right)$ obtained by each subject can reflect the dynamic brain effective connectivity network of the subject. The effective connection matrix of all the subjects was averaged to obtain the dynamic effective connectivity matrix (dECM, size: *m* × *m* × *n*) for each group.

(4) $$R_{w}\left(i,j\right)=\left\{\begin{array}{c} corr(X_{i,w},X_{j,w}),if\;i\neq j\\ 0,if\;\;i=j \end{array}\right.$$

By vectorizing the lower triangular elements in the functional connection matrix $R_{w}$ of each window, a dynamic FC intensity matrix $R_{\mathrm{total}}$ of $n\times \frac{m^{2}-m}{2}$ for each subject can be obtained. Each column of the matrix $R_{\mathrm{total}}$ represents the time‐varying changing trend between two brain regions (i.e., a specific dFC), and its variance is calculated to characterize the variability of each functional connection. Since there are ten FCs among five ROIs, we can get ten variance values, which form a vector for each subject. Obviously, the high consistency indicates that dFCs or dECs have a similar trend as time goes on. For example, when the dFC vector between V1 and V2 (denoted by dFC12) is highly correlated with dFC vector between V3 and V4 (dFC34), it is considered that the consistency of changing trend between dFC12 and dFC34 is high, that is, changing trend of different dFCs is similar. Similarly, the dynamic EC intensity matrix $F_{\mathrm{total}}$ (size: $n\times (m^{2}-m)$ ) is obtained, and twenty variance values are calculated for each subject.

**FIGURE 3:**
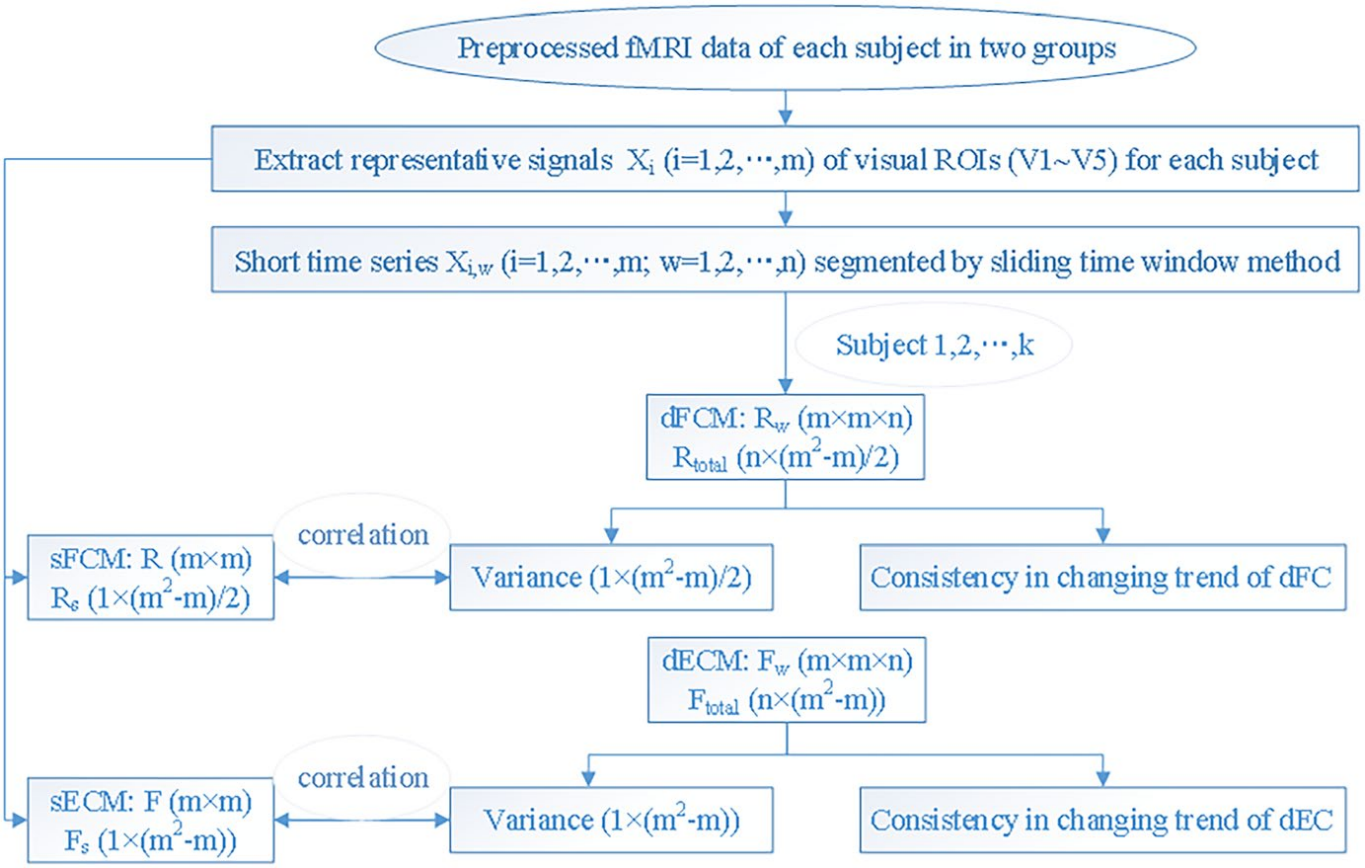
Overall processing flowchart. m is the number of ROIs; n is the number of windows; *k* is the number of subjects. dECM, static effective connection matrix; dFCM, dynamic functional connection matrix; sECM, static effective connection matrix; sFCM, static functional connection matrix

The overall processing flowchart is shown in Figure 3, which includes the above‐mentioned analyses.

## 3 RESULTS

### 3.1 Relationship between static and dynamic FCs

**FIGURE 4:**
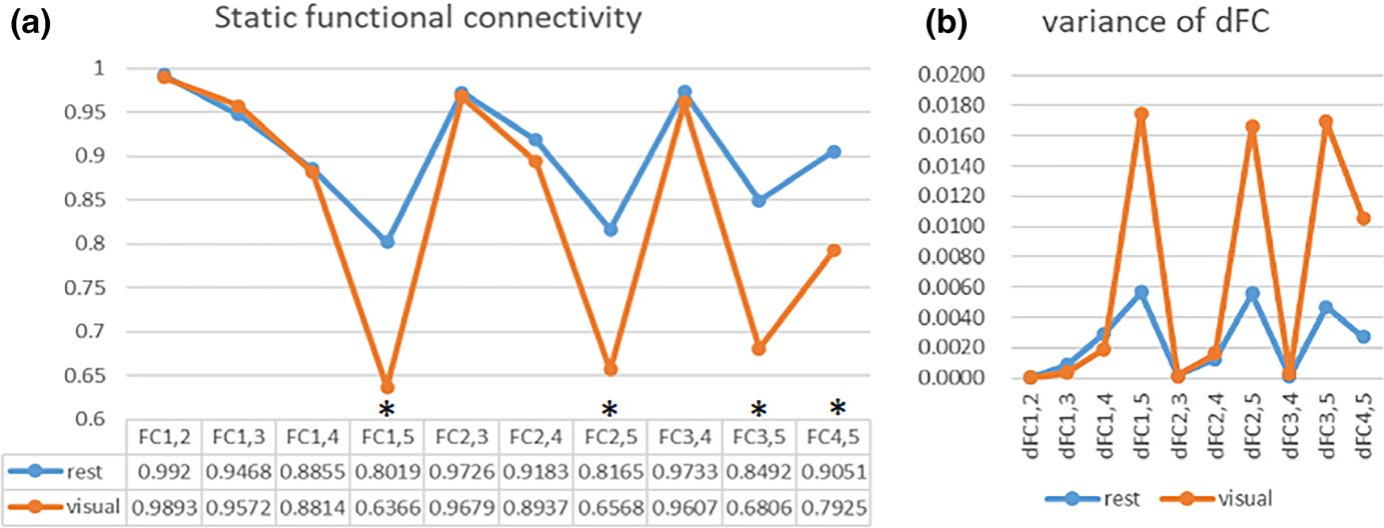
Mean sFC intensity (a) and dFC variance (b) for resting and task‐related states, respectively. ${\text{FC}}_{i,j}$ represents the Pearson correlation coefficient between time series of ROIi and ROIj. * denotes that there is a significant difference by independent‐sample *t* test (*p* < .005), indicating the functional connectivity between V5 and V1–V4 during visual stimulation is significantly lower than at rest

For the average sFC intensity ( $R_{s}$ ) and the average dFC variance of all subjects, the Pearson correlation coefficient between them was calculated. The result was −0.9867 for data at rest and −0.9841 for data recorded during a visual task. It shows that the sFC intensity and dFC variance are negatively correlated with each other. We can also see from Figure 4 that the average sFC intensity and the variance of dFC have opposite fluctuation trends regardless of the resting state or the visual stimulation state, that is, strong functional connectivity is always accompanied by small variability. After calculating Pearson correlation coefficient between sFC intensity and dFC variance likewise for each subject, we find the results were −0.8873 ± 0.1017 for 45 subjects at rest and −0.9245 ± 0.0798 during task, respectively. It also shows that there is a high negative correlation between sFC intensity and dFC variance as a whole. The larger the sFC intensity is, the smaller the variance of dFC would be. At level of single subject, the correlation was slightly reduced, which is likely due to individual differences or machine noise. We also used an independent‐sample *t* test to compare the differences in functional connectivity of the two groups for each pair of ROIs with threshold *p* < .005 (.05/10) correcting for multiple comparisons of correlations. FCs showing significant difference are denoted in Figure 4 with *. Detailed values of average sFC and variance of dFC are shown in Table S1, and statistical parameters of the difference in both states are shown in Table S3.

### 3.2 Relationship between static and dynamic ECs

**FIGURE 5:**
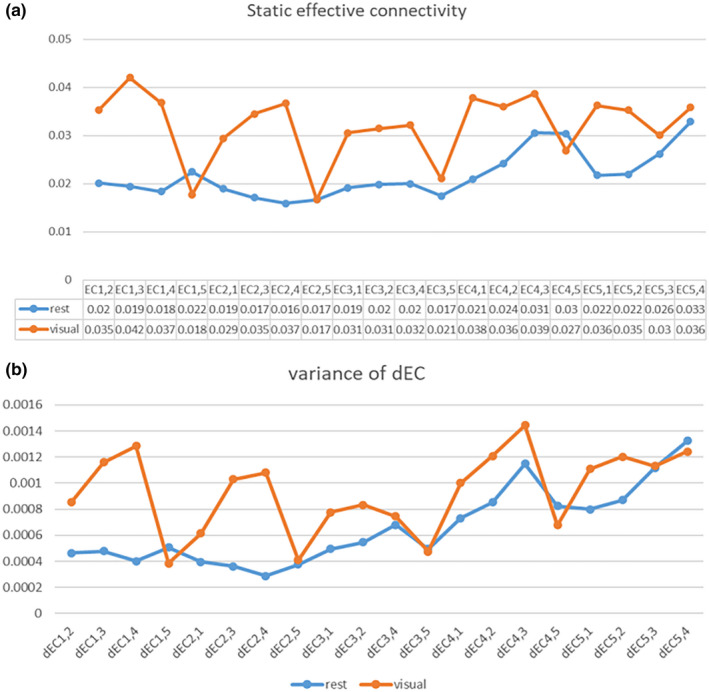
Mean sEC intensity (a) and dEC variance (b) for resting and task‐related states, respectively. ${\text{EC}}_{\mathrm{ij}}$ represents Granger causality from ROIi to ROIj, denoted by ROIi → ROIj

Likewise, for the average sEC intensity ( $F_{s}$ ) and the average dEC variance of all subjects, the Pearson correlation coefficient between them was calculated. The result was 0.8984 for data at rest and 0.8726 for data recorded during a visual task. We can observe that the average sEC intensity has a similar fluctuation trend with the dEC variance at rest and in the visual stimulation experiment. After calculating Pearson correlation coefficient between sEC intensity and dEC variance for each subject, we find the results were 0.6025 ± 0.2716 for 45 subjects at rest and 0.6634 ± 0.2675 during task, respectively. The Pearson correlation coefficient of the subject level is lower than that of group analysis. It is probably because causality value is small, and the group‐level calculation used the mean value of static connectivity intensity and variance of dynamic connectivity, which may balance out some individual differences. Overall, there is a high positive correlation between static EC intensity and dEC variance, and the larger the sEC intensity is, the larger the variance of the dEC variation would be. It can also be seen from Figure 5 that there are stronger effective connectivity and greater variability for data collected during visual stimulation than at rest though there is no significant difference in EC between two conditions (*p*> .05/20). Detailed values of average sEC and variance of dEC are shown in Table S2, and statistical parameters of the difference in both states are shown in Table S4. Besides, the results of other two dynamic measurement methods (i.e., ALFF and dispersion) of FC and EC fluctuation are demonstrated in Figure S1.

### 3.3 Consistency in changing trend of dFC

**FIGURE 6:**
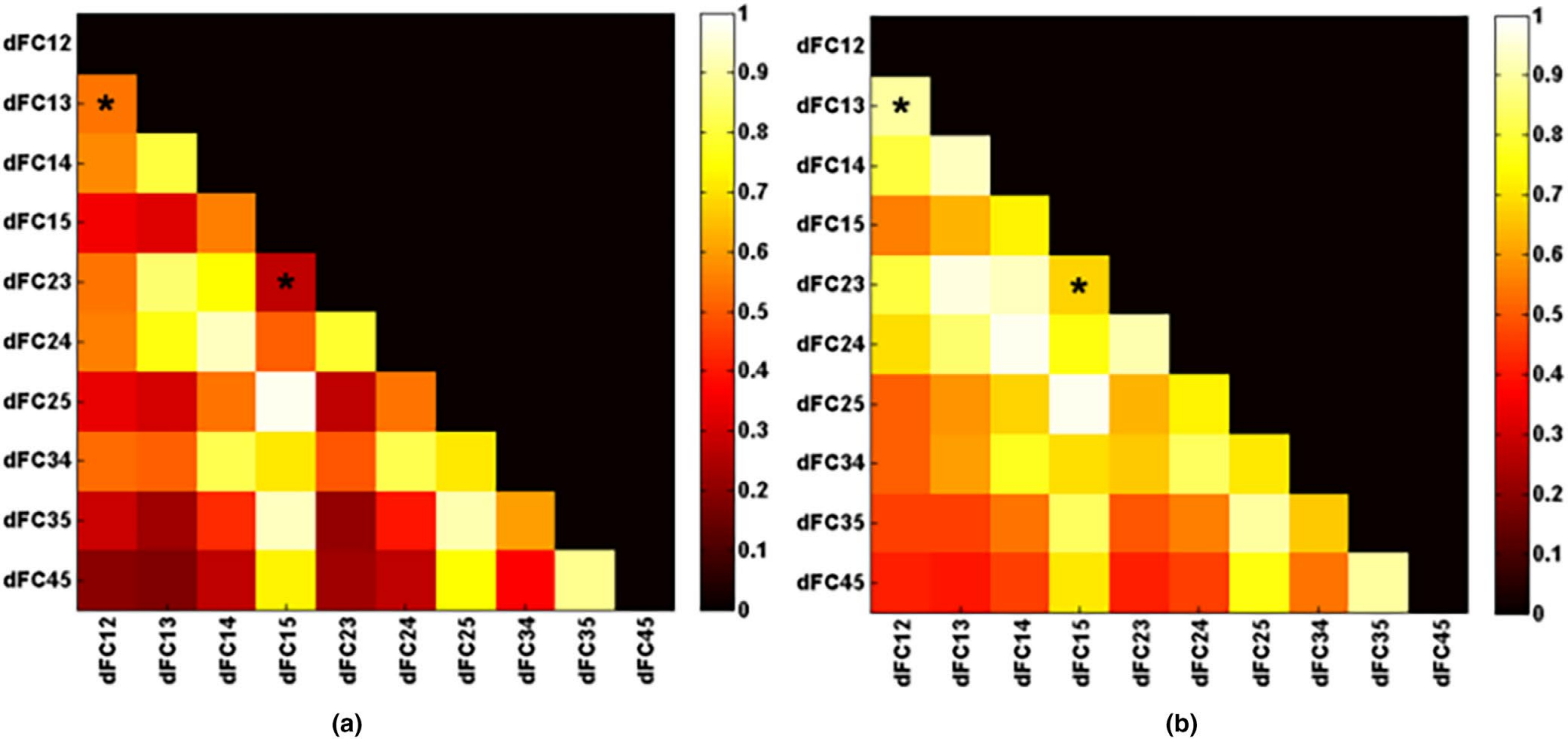
Average dFC correlation coefficient plots for visual stimulation experiment (a) and resting‐state experiment (b), respectively. * indicates significant difference in consistency of dFC changing trend by independent‐sample *t* test (*p* < .05/45). ${\text{dFC}}_{\mathrm{ij}}$ is a vector, which means time‐varying functional connectivity (namely, changing trend) with elements calculated by the Pearson correlation coefficient between ROIi and ROIj in all windows

Since the dFC or dEC between two certain brain regions can be viewed as a vector, which is described as the changing tread with elements calculated in all windows, the consistency of changing trend of all dFCs or dECs is studied to investigate the dynamic characteristics of brain connectivity. Each column of the dynamic functional connectivity strength matrix $R_{\text{total}}$ (*n* × 10) is the changing trend for each FC during an experiment, and each row is all FCs within a time window. The Pearson correlation coefficient among columns is calculated to obtain the correlation matrix (size: 10 × 10) for each subject, which is then averaged in each group (see Figure 6). It can be observed that the functional connectivity changes with similar trends. Compared to the resting state, the data during a visual stimulation showed a consistent decrease in changing trend between dFCs, which is denoted in Figure 6 with * (*p* < .0011, namely .05/45).

### 3.4 Consistency in changing trend of dEC

**FIGURE 7:**
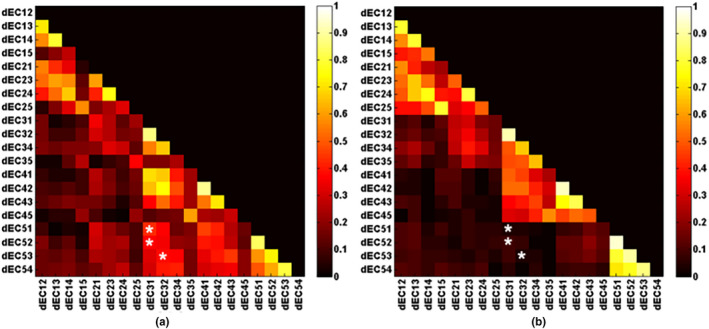
Average dEC correlation coefficient plots for visual stimulation experiment (a) and resting‐state experiment (b), respectively. * denotes significant differences in consistency of dEC changing trend by independent‐sample *t* test (*p* < .05/190). ${\text{dEC}}_{\mathrm{ij}}$ is a vector, which means time‐varying effective connectivity (namely, changing trend) from ROIi to ROIj in all windows with elements calculated by the Granger causality method

Each column of the dynamic effective connectivity strength matrix $F_{\text{total}}$ (*n* × 20) is the changing trend for each EC during an experiment, and each row is all ECs within a time window. The Pearson correlation coefficient among columns is calculated to obtain the correlation matrix (size: 20 × 20) for each subject. After taking the absolute value, the averaged correlation matrix is obtained (see Figure 7). Compared to the resting state, the task‐related data showed a consistent increase in changing trend between dEC, which is denoted in Figure 7 with * (*p* < 2.6316e−04, namely .05/190).

## 4 DISCUSSION

**FIGURE 8:**
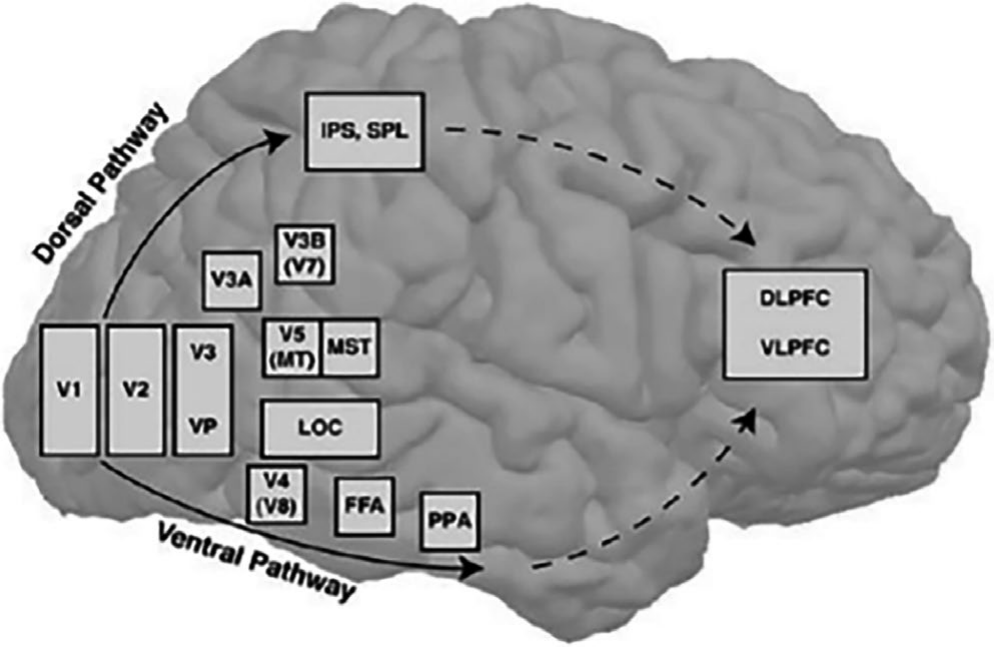
Dorsal and ventral pathways

Visual cortex is primarily responsible for visual information processing, which is located around the occipital lobe and receives visual information input from the lateral geniculate nucleus of the thalamus. The human visual cortex includes the primary visual cortex (V1, also known as the striate cortex) and the extrastriate cortex (such as V2, V3, V4, and V5). The flickering checkerboard stimulus experiment is the most widely used and stable method to explore the function of human brain visual system for clinical and scientific researchers engaged in ophthalmology and neuroscience. It provides complex visual stimuli, including optical and graphic information, so that the corresponding cortex of the subject can be significantly activated. Wohlschläger et al. (2005) studied the V1, V2, and Brodmann areas (BA) 17 and 18 of the functional magnetic resonance retinal brain map and found that they were basically consistent, respectively, indicating a certain degree of interoperability between functional partition and traditional anatomical partition. BA17 is the original sensory area that is directly subjected to visual stimuli and aims to identify the three‐dimensional structure of the object image such as form perception, depth perception, and color vision. BA18 and BA19, known as the visual association area, commonly used to synthesize visual information, form a conscious awareness and connect with motor, sensory, auditory, language, and other centers of ipsilateral and contralateral brains. The two‐stream hypothesis is a widely accepted and influential model of the neural processing of vision, which argues that humans possess two distinct visual systems (see Figure 8) (Ungerleider & Haxby, 1994). The dorsal stream (or, “where pathway”) stretches from the primary visual cortex (V1) in the occipital lobe forward into the parietal lobe and is proposed to be involved in the guidance of actions and recognize where objects are in space. Also known as “what pathway,” the ventral stream goes through V2 and V4 from V1 to areas of the inferior temporal lobe and is associated with object recognition and form representation. In the present paper, the visual cortex areas from V1 to V5 were selected for further analysis.

Recently, the temporal variability of functional connectivity and effective connectivity has attracted increasing attention (Park, Friston, Pae, Park, & Razi, 2018; Zalesky, Fornito, Cocchi, Gollo, & Breakspear, 2014). Functional brain networks demonstrate significant temporal variability and dynamic reconfiguration even in the resting state. Either sliding window or time–frequency analysis shows nonstationarity in spontaneous brain activity, which triggers temporal changes in connectivity of its functional architecture. As the resting state is an unconstrained condition that involves varying levels of mind‐wandering, arousal, attention, and vigilance, the temporal variability of functional brain networks derived from the BOLD‐fMRI may be driven ultimately by changes in mental state. In addition, specific changes in synchronization and information flow occur within and between networks that correlate with behavioral performance.

The temporal variability of a functional connectivity characterizes the changes in the Pearson correlation between BOLD signals of two corresponding ROIs. Low temporal variability means that the functional connectivity of two given ROIs is stable across different time windows, and vice versa. From Figure 4, we note the low variability, together with the strong functional connectivity within the visual network during task and rest states. It shows that whether in rest or task state, there are indeed widespread connectivities between brain regions in the visual cortex (Power, Schlaggar, & Petersen, 2014), and the network formed by the brain region is dynamic (Vidaurre et al., 2018). The human brain demonstrates tight association in its structure and function, and regions within one network tend to synchronize more easily with each other and thus have lower temporal variability. The results on FC variability are also in agreement with Ref. (Power et al., 2011), which suggested that visual system is rather stationary. It is meaningful and helpful to study static and dynamic connectivities at the same time, especially the relationship between them. Studies have shown that in different cognitive states, or different diseases, not only the connectivity of the brain changes, but also the variability of dynamic connectivity changes (Demirtaş et al., 2016). Sometimes, better classification characteristics than static characteristics can be obtained from dynamic brain connectivity analysis (Jie, Liu, & Shen, 2018; Qin et al., 2015). Fong et al. (2019) pointed out that combining static and dynamic FC features numerically improves predictions over either model alone. Incorporating dynamic FC features consistently improves predictions upon static FC alone and dFC may complement sFC in characterizing individual differences in attention. It figured out that static and dynamic matrices were highly dissimilar under both rest and task, but no specific relationship was explored. From Figure 4, we note that the sFC intensity has a strong negative correlation with variance of dFC, which is similar to previous studies. Deng, Sun, Cheng, and Tong (2016) discovered a strong negative correlation between inter‐regional FC and FC variability. Jin et al. (2017) found that PTSD subjects have stronger static connectivity, but reduced temporal variability of connectivity. Zhang et al. (2016) found that the temporal variability of a region correlates negatively with both the amplitude of its BOLD activity and the node degree, since the BOLD activity of a region and its degree are positively correlated. Thus, static and dynamic connectivities explore brain connectivity from different angles and comparing them within the context of the same study may help to better characterize the function of brain areas.

Compared with resting state, subjects in task state exhibited significantly decreased functional connectivity between V5 and V1–V4 (*p* < .005). The discovery that FCs among occipital lobe decrease during task state comparing with resting state is similar to previous studies (Cole, Bassett, Power, Braver, & Petersen, 2014; Spadone, et al., 2015). Comparisons of functional network connectivity during resting and task conditions also showed that functional network connectivity was stronger during rest compared to task (Arbabshirani, Havlicek, Kiehl, Pearlson, & Calhoun, 2013). According to Figure 6, the consistency of dFC changing trend in the visual stimulus state is smaller than rest state, indicating a little asynchronism in FC and providing evidence of smaller functional connectivity. One possibility of this effect is due to some difference in electrophysiological brain rhythms during resting state relative to task. For instance, alpha rhythms that are consistently present during rest may indirectly result in increased synchronizations in the BOLD signal, such that shifts to other frequencies during the flickering checkerboard condition decrease fMRI‐based FC compared with the fixation condition. Another possibility is that each brain region performs different functions in response to some aspects (not all aspects) of the task, thus causing increased activation and decreased synchronicity for respective responsibilities, which further explain the disassociation between FCs and BOLD response. Meanwhile, regions contributing significantly within a given functional area are often structurally connected to each other, or alternatively a brain region with more fiber connections to those of the same community would be involved more stably in that functional community, which will result in a strong connectivity. Thus, FCs between adjacent brain regions (such as V1 and V2, V2 and V3, V3 and V4, V4 and V5) are relatively larger than remote brain regions (such as V1 and V5) and show less variability. Also, there is evidence that middle/superior occipital gyrus demonstrates low variability, while middle temporal gyrus demonstrates a high variability, which may also explain why variabilities of FC among V1–V4 are smaller than those involving V5 (Zhang et al., 2016).

As with the temporal variability of FC, the temporal variability of EC is defined as the variance of dynamic EC in all time windows across the whole experiment. That is, the fluctuation amplitude of the Granger causality time courses represents the variability of each connection between regions over time. As far as we know, no fMRI studies have focused on the relationship between static and dynamic ECs. However, it is discovered that static EC is positively correlated with variance of the dEC, which is different from the relationship in FC and is novel to our perception. It means that large effective connectivity is accompanied by large variance of dEC. We speculated that when the brain receives visual stimulation, the information flows in the visual cortex changes and keeps at high level for a period of time. After the visual simulation disappears and the screen reverts to black, the information flow returns back to the baseline state. So, the changes in information flow in brain regions may be due to the cyclical changes in external stimuli during a block‐designed experiment. The more ECs among V1–V5 increase when receiving continuous visual stimulation, the more they differ with resting state, which will result in larger variability as the ECs need to increase and recover to resting level periodically.

There is no significant difference in effective connectivity between two groups (*p*> .05/20) though we can find that the ECs among V1–V5 increase during task state compared with resting state when using a less stringent correction threshold especially EC12, EC13, EC14, EC21, and EC23 (see Figure 5). This phenomenon of increase in EC among V1–V5 is consistent with visual formation as the visual cortex produces the flow of information when stimulated. It is generally believed that V2 and V3 revolve around V1 and accept the contact fibers emitted by V1. They are not limited to a certain function, but process and integrate various information to complete advanced cognitive activity. V2 is the second major visual area of the visual cortex and the first station of the visual association area, receiving strong feedforward connection from V1, and sending connection to V3–V5, and also having strong feedback connection to V1. V3 is located in the front of V2, equivalent to anatomically Brodmann area 19, which receives input from V1 and V2 and is projected into the posterior parietal cortex. The dorsal and ventral parts of V3 are responsible for the lower and upper 1/4 of the lateral field of vision, respectively. V4 is the third visual area of the ventral stream, receiving powerful feedforward input from V2. V4 also receives direct input from V1, especially the central part. Similar to V1, V4 modulates orientation, spatial frequency, and color stimuli, which are just included in a flickering checkerboard, but it can only modulate moderately complex features of objects, such as simple geometric shapes of objects, and cannot process information about complex objects like faces. It can also be reflected from Figure 5a that the effective connectivity between V4 and other visual areas is larger than that of the resting state. The V5 region, also known as the middle temporal gyrus, is composed of many neurons that are selective to the movement of complex visual stimuli, which can integrate local visual signals into the overall movement of complex objects. In this paper, the flickering checkerboard visual stimulation experiment did not contain much information about motion, so the dorsal stream through V5 did not change significantly. Figure 7 shows an increase in consistency of changing trend among dEC during visual stimulus state, which indicates that the EC shows stronger synchronization in different windows, that is, EC has similar changing trend, which may explain why it is stronger than that at rest in some aspects.

**FIGURE 9:**
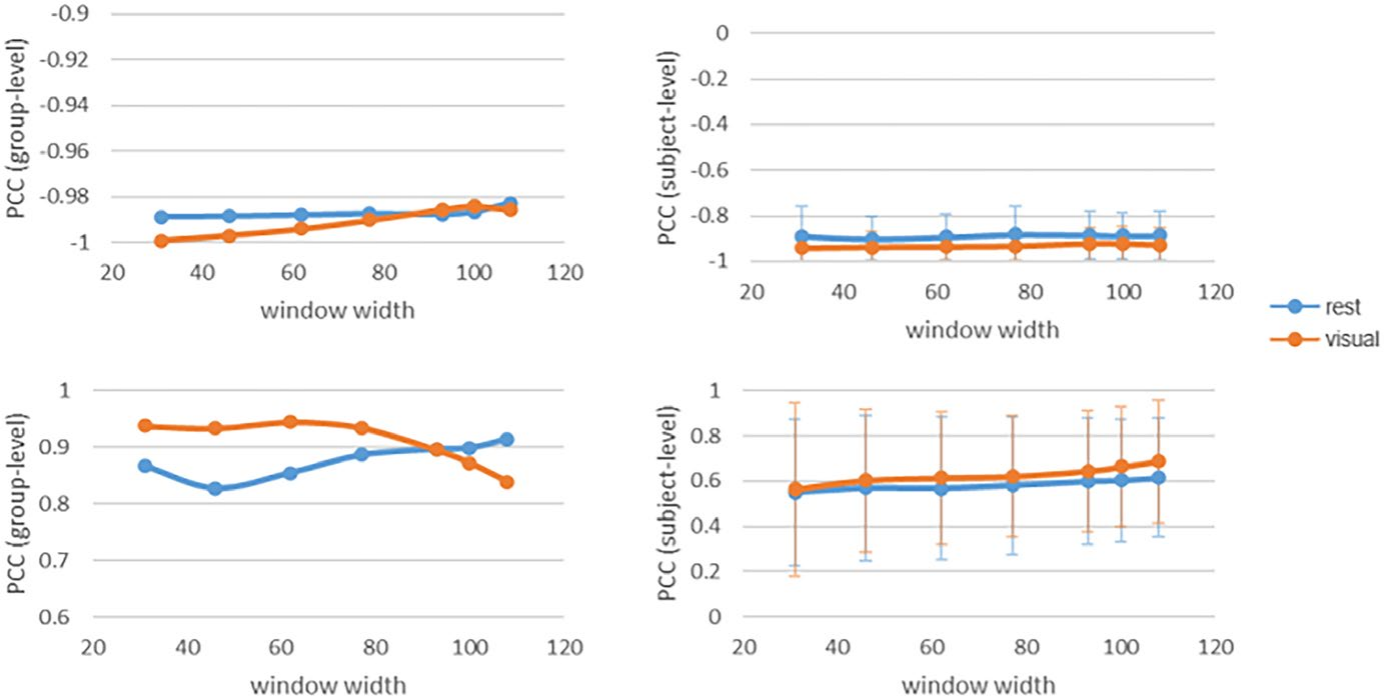
Pearson correlation coefficient between variance of dynamic FC and intensity of sFC (a) and variance of dynamic EC and intensity of sEC (b) under different window width from group level and subject level, respectively

Choosing an appropriate window size is an area of concern when using the sliding window approach to estimate FC and EC dynamics. Theoretically, the window size should be sufficiently small enough to detect potentially interesting transients in the low‐frequency fluctuations in brain connectivity. However, an excessively small window will decrease the signal‐to‐noise ratio (SNR) of the estimated dFC and dEC. Since the duration of design block of the experimental paradigm is 40 s (62 × 0.645 s), the window width is set to 31, 46, 62, 77, 93, 100, and 108, respectively, to measure the impact of the sliding window size on relationship between static and dynamic connectivities. Seven different window widths were employed to calculate the Pearson correlation coefficient between intensity of static connectivity and variance of dynamic connectivity. The result demonstrated that the influence of window size on PCC results was relatively minimal (see Figure 9). Besides, the results of the other two dynamic measurement methods (i.e., ALFF and dispersion) are illustrated in Figures S2 and S3.

## 5 CONCLUSION

fMRI has the characteristics of real‐time and high spatial–temporal resolution, and has been widely used in the basic cognitive research and clinic of ophthalmic fields such as optic nerve disease and ophthalmic acupuncture treatment. The present study explores time‐varying coupling and causal information of the modulation effects among several subareas of human visual cortex (V1–V5) (Samdin, Ting, Salleh, Hamedi, & Noor, 2016; Xin & Biswal, 2015). Besides, the relationship between static and dynamic connectivities especially for static EC (sEC) and dynamic EC (dEC), as well as the consistency characteristics of changing trend of dFCs and dECs, is also investigated. The connection intensity and information flow were calculated in each window among the visual areas by the Pearson correlation coefficient and Granger causality analysis, respectively, over time with sliding window method. The results demonstrate that there are extensive connections existing in human visual network, which are time‐varying both in resting and task‐related states. sFC intensity is negatively correlated with the variance of dFC, while sEC intensity is positively correlated with the variance of dEC. Furthermore, we also find that dFC within visual cortex at rest shows more consistency, while dEC shows less compared with task state in changing trend. Therefore, this study provides insights into the dynamics of connectivity in human visual cortex and the changes in visual pathways when visually stimulated from the perspective of functional and effective connectivities. This may contribute to the study of the clinical diagnosis, treatment, and pathological mechanism research of ophthalmic diseases such as amblyopia, which is caused by the lack of effective stimulation of visual cells due to various reasons during visual development in terms of diagnosis and treatment.

## 6 IMPLICATIONS AND FUTURE STUDIES

Still, there are three implications that need further study. First, this paper studies the dynamic changes in FC and EC in visual cortex during a visual task and the comparison with resting state. However, it is impossible to determine the exact neuronal mechanisms in the brain that are subject to changes in task modulation. For example, this may be due to short‐term brain plasticity regulation (Yao, Shi, Han, Gao, & Dan, 2007), or synchronous oscillations in neural cell clusters (Buzsáki & Draguhn, 2004). A single brain imaging technique can lead to incomplete information acquisition. In the future, multimodal data such as PET, MEG, and EEG can be combined to obtain more information on brain activity. Second, the brain is an organic whole and there are wide connections across the brain particularly between the visual area and other brain functional areas such as BA39 area, which involves spatial imagination and visual movement, BA7 area, which refers to temporal and spatial processing and memory retrieval, BA37 area, which relates to vision and language (vocabulary and object recognition, naming and face recognition). This study only selected visual subareas V1–V5 as the research object and the characteristics of dynamic connectivity under visual stimulus task can be examined from whole brain in the future. Third, the Pearson correlation, Granger causality analysis, and sliding time window method are used as measurement methods for functional connectivity, effective connectivity, and dynamics assessment (Calhoun & Adali, 2016; Thompson & Fransson, 2017) in this paper. In the future, other measurement methods can be used to further verify the experimental results of this paper.

## CONFLICT OF INTEREST

All authors declare no conflict of interest.

## AUTHOR CONTRIBUTIONS

LZ and WZ conceived and designed the study. LZ analyzed the data and wrote the article. WN and YS advised on the proposed method. WZ, YS, and JY made the critical revision of the article. All authors reviewed the manuscript.

## Supporting information

Supplementary Material

## Data Availability

The data that support the findings of this study are available from the corresponding author upon reasonable request.

## References

[brb31698-bib-0001] Allen, E. A. , Damaraju, E. , Plis, S. M. , Erhardt, E. B. , Eichele, T. , & Calhoun, V. D. (2014). Tracking whole‐brain connectivity dynamics in the resting state. Cerebral Cortex, 24(3), 663–676. 23146964 10.1093/cercor/bhs352 PMC3920766

[brb31698-bib-0002] Arbabshirani, M. R. , Havlicek, M. , Kiehl, K. A. , Pearlson, G. D. , & Calhoun, V. D. (2013). Functional network connectivity during rest and task conditions: A comparative study. Human Brain Mapping, 34(11), 2959–2971. 22736522 10.1002/hbm.22118 PMC3524389

[brb31698-bib-0003] Bassett, D. S. , Wymbs, N. F. , Porter, M. A. , Mucha, P. J. , Carlson, J. M. , & Grafton, S. T. (2011). Dynamic reconfiguration of human brain networks during learning. Proceedings of the National Academy of Sciences of the United States of America, 108(18), 7641–7646. 10.1073/pnas.1018985108 21502525 PMC3088578

[brb31698-bib-0004] Bavelier, D. , Tomann, A. , Hutton, C. , Mitchell, T. , Corina, D. , Liu, G. , & Neville, H. (2000). Visual attention to the periphery is enhanced in congenitally deaf individuals. The Journal of Neuroscience, 20(17), RC93 10.1523/JNEUROSCI.20-17-j0001.2000 10952732 PMC6772982

[brb31698-bib-0005] Belliveau, J. W. , Kennedy Jr, D. N. , McKinstry, R. C. , Buchbinder, B. R. , Weisskoff, R. M. , Cohen, M. S. , … Rosen, B. R. (1991). Functional mapping of the human visual cortex by magnetic resonance imaging. Science, 254(5032), 716–719. 1948051 10.1126/science.1948051

[brb31698-bib-0006] Buzsáki, G. , & Draguhn, A. (2004). Neuronal oscillations in cortical networks. Science, 304(5679), 1926 10.1126/science.1099745 15218136

[brb31698-bib-0007] Calhoun, V. D. , & Adali, T. (2016). Time‐varying brain connectivity in fMRI data: Whole‐brain data‐driven approaches for capturing and characterizing dynamic states. IEEE Signal Processing Magazine, 33(3), 52–66. 10.1109/MSP.2015.2478915

[brb31698-bib-0008] Cole, M. W. , Bassett, D. S. , Power, J. D. , Braver, T. S. , & Petersen, S. E. (2014). Intrinsic and task‐evoked network architectures of the human brain. Neuron, 83(1), 238–251. 10.1016/j.neuron.2014.05.014 24991964 PMC4082806

[brb31698-bib-0009] Demirtaş, M. , Tornador, C. , Falcon, C. , López‐Solà, M. , Hernández‐Ribas, R. , Pujol, J. , … Deco, G. (2016). Dynamic functional connectivity reveals altered variability in functional connectivity among patients with major depressive disorder. Human Brain Mapping, 37(8), 2918–2930. 27120982 10.1002/hbm.23215 PMC5074271

[brb31698-bib-0010] Deng, L. , Sun, J. , Cheng, L. , & Tong, S. (2016). Characterizing dynamic local functional connectivity in the human brain. Scientific Reports, 6, 26976. 27231194 10.1038/srep26976 PMC4882585

[brb31698-bib-0011] Di, X. , Fu, Z. , Chan, S. C. , Hung, Y. S. , Biswal, B. B. , & Zhang, Z. (2015). Task‐related functional connectivity dynamics in a block‐designed visual experiment. Frontiers in Human Neuroscience, 9, 543 10.3389/fnhum.2015.00543 26483660 PMC4588125

[brb31698-bib-0012] Dimitriadis, S. I. , Laskaris, N. A. , Tsirka, V. , Vourkas, M. , & Micheloyannis, S. (2012). An eeg study of brain connectivity dynamics at the resting state. Nonlinear Dynamics Psychology & Life Sciences, 16(1), 5. 22196109

[brb31698-bib-0013] Eickhoff, S. B. , Stephan, K. E. , Mohlberg, H. , Grefkes, C. , Fink, G. R. , Amunts, K. , & Zilles, K. (2005). A new spm toolbox for combining probabilistic cytoarchitectonic maps and functional imaging data. NeuroImage, 25(4), 1325–1335. 10.1016/j.neuroimage.2004.12.034 15850749

[brb31698-bib-0014] Faes, L. , Nollo, G. , Stramaglia, S. , & Marinazzo, D. (2017). Multiscale granger causality. Physical Review E, 96(4), 042150 10.1103/PhysRevE.96.042150 29347576

[brb31698-bib-0015] Fong, A. H. , Yoo, K. , Rosenberg, M. D. , Zhang, S. , Li, C. S. , Scheinost, D. , … Chun, M. M. (2019). Dynamic functional connectivity during task performance and rest predicts individual differences in attention across studies. NeuroImage, 188, 14–25. 10.1016/j.neuroimage.2018.11.057 30521950 PMC6401236

[brb31698-bib-0016] Friston, K. J. , Frith, C. D. , & Frackowiak, R. S. J. (1993). Time‐dependent changes in effective connectivity measured with PET. Human Brain Mapping, 1(1), 69–79. 10.1002/hbm.460010108

[brb31698-bib-0017] Gao, L. , Sommerlade, L. , Coffman, B. , Zhang, T. , Stephen, J. M. , Li, D. , … Schelter, B. (2015). Granger causal time‐dependent source connectivity in the somatosensory network. Scientific Reports, 5, 10399 10.1038/srep10399 25997414 PMC4441010

[brb31698-bib-0018] Glasser, M. F. , Coalson, T. S. , Robinson, E. C. , Hacker, C. D. , Harwell, J. , Yacoub, E. , … Van Essen, D. C. (2016). A multi‐modal parcellation of human cerebral cortex. Nature, 536(7615), 171–178. 27437579 10.1038/nature18933 PMC4990127

[brb31698-bib-0019] Gonzalez‐Castillo, J. , & Bandettini, P. A. (2017). Task‐based dynamic functional connectivity: Recent findings and open questions. NeuroImage, 180(Pt B), 526–533. 28780401 10.1016/j.neuroimage.2017.08.006 PMC5797523

[brb31698-bib-0020] Hu, L. , Zhang, Z. G. , & Hu, Y. (2012). A time‐varying source connectivity approach to reveal human somatosensory information processing. NeuroImage, 62(1), 217–228. 10.1016/j.neuroimage.2012.03.094 22580382

[brb31698-bib-0021] Hutchison, R. M. , Womelsdorf, T. , Allen, E. A. , Bandettini, P. A. , Calhoun, V. D. , Corbetta, M. , … Chang, C. (2013). Dynamic functional connectivity: Promise, issues, and interpretations. NeuroImage, 80(1), 360 10.1016/j.neuroimage.2013.05.079 23707587 PMC3807588

[brb31698-bib-0022] Hutchison, R. M. , Womelsdorf, T. , Gati, J. S. , Everling, S. , & Menon, R. S. (2013). Resting‐state networks show dynamic functional connectivity in awake humans and anesthetized macaques. Human Brain Mapping, 34(9), 2154–2177. 10.1002/hbm.22058 22438275 PMC6870538

[brb31698-bib-0023] Jie, B. , Liu, M. , & Shen, D. (2018). Integration of temporal and spatial properties of dynamic connectivity networks for automatic diagnosis of brain disease. Medical Image Analysis, 47, 81–94. 10.1016/j.media.2018.03.013 29702414 PMC5986611

[brb31698-bib-0024] Jin, C. , Jia, H. , Lanka, P. , Rangaprakash, D. , Li, L. , Liu, T. , … Deshpande, G. (2017). Dynamic brain connectivity is a better predictor of PTSD than static connectivity. Human Brain Mapping, 38(9), 4479–4496. 10.1002/hbm.23676 28603919 PMC6866943

[brb31698-bib-0025] Kaiser, R. H. , Whitfieldgabrieli, S. , Dillon, D. G. , Goer, F. , Beltzer, M. , Minkel, J. , … & Pizzagalli, D. A. (2016). Dynamic resting‐state functional connectivity in major depression. Neuropsychopharmacology, 41(7), 1822–1830. 10.1038/npp.2015.352 26632990 PMC4869051

[brb31698-bib-0026] Logothetis, N. K. , Pauls, J. , Augath, M. , Trinath, T. , & Oeltermann, A. (2001). Neurophysiological investigation of the basis of the fMRI signal. Nature, 412(6843), 150 10.1038/35084005 11449264

[brb31698-bib-0027] Luo, C. , Yang, F. , Deng, J. , Zhang, Y. , Hou, C. , Huang, Y. , … Yao, D. (2016). Altered functional and effective connectivity in anticorrelated intrinsic networks in children with benign childhood epilepsy with centrotemporal spikes. Medicine, 95(24), e3831 10.1097/MD.0000000000003831 27310959 PMC4998445

[brb31698-bib-0028] Nooner, K. B. , Colcombe, S. J. , Tobe, R. H. , Mennes, M. , & Milham, M. P. (2012). The nki‐rockland sample: A model for accelerating the pace of discovery science in psychiatry. Frontiers in Neuroscience, 6(152), 152 10.3389/fnins.2012.00152 23087608 PMC3472598

[brb31698-bib-0029] Park, H. J. , Friston, K. , Pae, C. , Park, B. , & Razi, A. (2018). Dynamic effective connectivity in resting state fMRI. NeuroImage, 180(Pt B), 594–608. 29158202 10.1016/j.neuroimage.2017.11.033 PMC6138953

[brb31698-bib-0030] Power, J. D. , Cohen, A. L. , Nelson, S. M. , Wig, G. S. , Barnes, K. A. , Church, J. A. , … Petersen, S. E. (2011). Functional network organization of the human brain. Neuron, 72(4), 665 10.1016/j.neuron.2011.09.006 22099467 PMC3222858

[brb31698-bib-0031] Power, J. , Schlaggar, B. , & Petersen, S. (2014). Studying brain organization via spontaneous fMRI signal. Neuron, 84(4), 681–696. 10.1016/j.neuron.2014.09.007 25459408 PMC4254503

[brb31698-bib-0032] Qin, J. , Chen, S. G. , Hu, D. , Zeng, L. L. , & Shen, H. (2015). Predicting individual brain maturity using dynamic functional connectivity. Frontiers in Human Neuroscience, 9(10), 418. 26236224 10.3389/fnhum.2015.00418 PMC4503925

[brb31698-bib-0033] Samdin, S. B. , Ting, C. M. , Salleh, S. H. , Hamedi, M. , & Noor, A. M. (2016). Identifying dynamic effective connectivity states in fMRI based on time‐varying vector autoregressive models. International Conference for Innovation in Biomedical Engineering and Life Sciences. Singapore City, Singapore: Springer.

[brb31698-bib-0034] Sereno, M. I. , Dale, A. M. , Reppas, J. B. , Kwong, K. K. , Belliveau, J. W. , Brady, T. J. , … Tootell, R. B. H. (1995). Borders of multiple visual areas in humans revealed by functional mri. Science, 268(5212), 889–893. 7754376 10.1126/science.7754376

[brb31698-bib-0035] Shakil, S. , Lee, C. H. , & Keilholz, S. D. (2016). Evaluation of sliding window correlation performance for characterizing dynamic functional connectivity and brain states. NeuroImage, 133, 111–128. 10.1016/j.neuroimage.2016.02.074 26952197 PMC4889509

[brb31698-bib-0036] Shi, Y. , Zeng, W. , Wang, N. , & Chen, D. (2015). A novel fmri group data analysis method based on data‐driven reference extracting from group subjects. Computer Methods and Programs in Biomedicine, 122(3), 362–371. 26387634 10.1016/j.cmpb.2015.09.002

[brb31698-bib-0037] Shi, Y. , Zeng, W. , Wang, N. , & Zhao, L. (2018). A new constrained spatiotemporal ICA method based on multi‐objective optimization for fMRI data analysis. IEEE Transactions on Neural Systems & Rehabilitation Engineering, 26(9), 1690–1699. 30028710 10.1109/TNSRE.2018.2857501

[brb31698-bib-0038] Spadone, S. , Della, P. S. , Sestieri, C. , Betti, V. , Tosoni, A. , Perrucci, M. G. , … Corbetta, M. (2015). Dynamic reorganization of human resting‐state networks during visuospatial attention. Proceedings of the National Academy of Sciences of the United States of America, 112(26), 8112–8117. 10.1073/pnas.1415439112 26080395 PMC4491799

[brb31698-bib-0039] Thompson, W. H. , & Fransson, P. (2017). A common framework for the problem of deriving estimates of dynamic functional brain connectivity. NeuroImage, 172, 896. 29292136 10.1016/j.neuroimage.2017.12.057

[brb31698-bib-0040] Tian, L. , Jiang, T. , Liang, M. , Li, X. , He, Y. , Wang, K. , … Jiang, T. (2010). Stabilities of negative correlations between blood oxygen level‐dependent signals associated with sensory and motor cortices. Human Brain Mapping, 28(7), 681–690. 10.1002/hbm.20300 PMC6871341 17266102

[brb31698-bib-0041] Tian, L. , Li, Q. , Wang, C. , & Yu, J. (2018). Changes in dynamic functional connections with aging. NeuroImage, 172, 31–39. 10.1016/j.neuroimage.2018.01.040 29414496

[brb31698-bib-0042] Tobia, M. J. , Hayashi, K. , Ballard, G. , Gotlib, I. H. , & Waugh, C. E. (2017). Dynamic functional connectivity and individual differences in emotions during social stress. Human Brain Mapping, 38(12), 6185–6205. 10.1002/hbm.23821 28940859 PMC6866845

[brb31698-bib-0043] Ungerleider, L. G. , & Haxby, J. V. (1994). ‘What’ and ‘where’ in the human brain. Current Opinion in Neurobiology, 4(2), 157–165. 10.1016/0959-4388(94)90066-3 8038571

[brb31698-bib-0044] Vicente, R. , Wibral, M. , Lindner, M. , & Pipa, G. (2011). Transfer entropy—A model‐free measure of effective connectivity for the neurosciences. Journal of Computational Neuroscience, 30(1), 45–67. 10.1007/s10827-010-0262-3 20706781 PMC3040354

[brb31698-bib-0045] Vidaurre, D. , Abeysuriya, R. , Becker, R. , Quinn, A. J. , Alfaro‐Almagro, F. , Smith, S. M. , & Woolrich, M. W. (2018). Discovering dynamic brain networks from big data in rest and task. NeuroImage, 180(Pt B), 646–656. 10.1016/j.neuroimage.2017.06.077 28669905 PMC6138951

[brb31698-bib-0046] Warnking, J. , Dojat, M. , Guérin‐Dugué, A. , Delon‐Martin, C. , Olympieff, S. , Richard, N. , … Segebarth, C. (2002). fMRI retinotopic mapping–step by step. NeuroImage, 17(4), 1665–1683. 10.1006/nimg.2002.1304 12498741

[brb31698-bib-0047] Wohlschläger, A. M. , Specht, K. , Lie, C. , Mohlberg, H. , Wohlschläger, A. , Bente, K. , … Fink, G. R. (2005). Linking retinotopic fmri mapping and anatomical probability maps of human occipital areas v1 and v2. NeuroImage, 26(1), 73–82. 10.1016/j.neuroimage.2005.01.021 15862207

[brb31698-bib-0048] Xin, D. , & Biswal, B. B. (2014). Identifying the default mode network structure using dynamic causal modeling on resting‐state functional magnetic resonance imaging. NeuroImage, 86(2), 53–59. 10.1016/j.neuroimage.2013.07.071 23927904 PMC3947265

[brb31698-bib-0049] Xin, D. , & Biswal, B. B. (2015). Dynamic brain functional connectivity modulated by resting‐state networks. Brain Structure & Function, 220(1), 37–46. 10.1007/s00429-013-0634-3 25713839 PMC3980132

[brb31698-bib-0050] Yan, C. C. , & Zang, Y. F. (2010). Dparsf: A matlab toolbox for “pipeline” data analysis of resting‐state fMRI. Frontiers in Systems Neuroscience, 4(13), 13 10.3389/fnsys.2010.00013 20577591 PMC2889691

[brb31698-bib-0051] Yao, H. , Shi, L. , Han, F. , Gao, H. , & Dan, Y. (2007). Rapid learning in cortical coding of visual scenes. Nature Neuroscience, 10(6), 772 10.1038/nn1895 17468750

[brb31698-bib-0052] Zalesky, A. , Fornito, A. , Cocchi, L. , Gollo, L. L. , & Breakspear, M. (2014). Time‐resolved resting‐state brain networks. Proceedings of the National Academy of Sciences of the United States of America, 111(28), 10341–10346. 10.1073/pnas.1400181111 24982140 PMC4104861

[brb31698-bib-0053] Zhang, J. , Cheng, W. , Liu, Z. , Zhang, K. , Lei, X. , Yao, Y. , … Feng, J. (2016). Neural, electrophysiological and anatomical basis of brain‐network variability and its characteristic changes in mental disorders. Brain, 139(Pt 8), 2307–2321. 10.1093/brain/aww143 27421791

[brb31698-bib-0054] Zhang, X. , Guo, L. , Li, X. , Zhang, T. , Zhu, D. , Li, K. , … Li, L. (2013). Characterization of task‐free and task‐performance brain states via functional connectome patterns. Medical Image Analysis, 17(8), 1106–1122. 23938590 10.1016/j.media.2013.07.003 PMC3956081

[brb31698-bib-0055] Zhou, J. , & Zinai, L. I. (2004). Applicability of the granger causality test. Journal of Tsinghua University, 3, 358–361.

