# Supplementary material for: Dynamic visual cortical connectivity analysis based on functional magnetic resonance imaging
Source: Brain Behav. 2020 Jun 7;10(7):e01698. doi: 10.1002/brb3.1698 (PMC7375061; doi:10.1002/brb3.1698)
Supplement: Supplementary file 1 — Supplementary Material [file BRB3-10-e01698-s001.docx]

**Supplemental Materials**

**Supplemental Table 1** Average static FC and variance of dFC with the window width set to 100. FC_i,j_ represents the Pearson correlation coefficient between time series of ROIi and ROIj. sFC, static functional connectivity; dFC, dynamic functional connectivity; PCC, Pearson correlation coefficient.

|  | Rest | | Visual | |
| --- | --- | --- | --- | --- |
|  | Average sFC | Variance of dFC | Average sFC | Variance of dFC |
| FC_1,2_ | 0.9920 | 0.0001 | 0.9893 | 0.0000 |
| FC_1,3_ | 0.9468 | 0.0009 | 0.9572 | 0.0004 |
| FC_1,4_ | 0.8855 | 0.0029 | 0.8814 | 0.0019 |
| FC_1,5_ | 0.8019 | 0.0057 | 0.6366 | 0.0175 |
| FC_2,3_ | 0.9726 | 0.0002 | 0.9679 | 0.0002 |
| FC_2,4_ | 0.9183 | 0.0012 | 0.8937 | 0.0016 |
| FC_2,5_ | 0.8165 | 0.0056 | 0.6568 | 0.0166 |
| FC_3,4_ | 0.9733 | 0.0001 | 0.9607 | 0.0003 |
| FC_3,5_ | 0.8492 | 0.0047 | 0.6806 | 0.0169 |
| FC_4,5_ | 0.9051 | 0.0027 | 0.7925 | 0.0106 |
| PCC  (Group-level) | -0.9867 | | -0.9841 | |
| PCC  (Subject-level) | -0.8873±0.1017 | | -0.9245±0.0798 | |

**Supplemental Table 2** Average static EC and variance of dEC with the window width set to 100. EC_i,j_ represents Granger causality from ROIi to ROIj, denoted by ROIi→ROIj. sEC, static effective connectivity; dEC, dynamic effective connectivity.

|  | Rest | | Visual | |
| --- | --- | --- | --- | --- |
|  | Average sEC | Variance of dEC | Average sEC | Variance of dEC |
| EC_1,2_ | 0.0201 | 0.0005 | 0.0352 | 0.0006 |
| EC_1,3_ | 0.0194 | 0.0005 | 0.0420 | 0.0010 |
| EC_1,4_ | 0.0183 | 0.0004 | 0.0368 | 0.0011 |
| EC_1,5_ | 0.0224 | 0.0005 | 0.0177 | 0.0004 |
| EC_2,1_ | 0.0189 | 0.0004 | 0.0294 | 0.0008 |
| EC_2,3_ | 0.0171 | 0.0004 | 0.0345 | 0.0008 |
| EC_2,4_ | 0.0159 | 0.0003 | 0.0367 | 0.0007 |
| EC_2,5_ | 0.0167 | 0.0004 | 0.0167 | 0.0005 |
| EC_3,1_ | 0.0191 | 0.0005 | 0.0305 | 0.0010 |
| EC_3,2_ | 0.0199 | 0.0005 | 0.0314 | 0.0012 |
| EC_3,4_ | 0.0200 | 0.0007 | 0.0322 | 0.0014 |
| EC_3,5_ | 0.0174 | 0.0005 | 0.0210 | 0.0007 |
| EC_4,1_ | 0.0209 | 0.0007 | 0.0378 | 0.0011 |
| EC_4,2_ | 0.0242 | 0.0009 | 0.0360 | 0.0012 |
| EC_4,3_ | 0.0305 | 0.0011 | 0.0387 | 0.0011 |
| EC_4,5_ | 0.0304 | 0.0008 | 0.0269 | 0.0012 |
| EC_5,1_ | 0.0217 | 0.0008 | 0.0362 | 0.0006 |
| EC_5,2_ | 0.0219 | 0.0009 | 0.0353 | 0.0010 |
| EC_5,3_ | 0.0262 | 0.0011 | 0.0300 | 0.0011 |
| EC_5,4_ | 0.0329 | 0.0013 | 0.0358 | 0.0004 |
| PCC  (Group-level) | 0.8984 | | 0.8726 | |
| PCC  (Subject-level) | 0.6025±0.2716 | | 0.6634±0.2675 | |

**Supplemental Table 3** Significantly different connections based on static FC (sFC) and variability of FC (vFC). The window width is set to 100 for constructing dynamic FC. ** denotes that there is a significant difference by independent sample t test (p < 0.05/10) and * denotes that there is a difference by independent sample t test (p < 0.05).

| Static functional connectivity (sFC) | | |  | Variability of functional connectivity (vFC) | | |
| --- | --- | --- | --- | --- | --- | --- |
| Pair | T-statistic | P |  | Pair | T-statistic | P |
| FC_1,5_ ^**^ | 4.6027 | 0.000017 |  |  |  |  |
| FC_2,5_ ^**^ | 4.7089 | 0.000011 |  |  |  |  |
| FC_3,5_ ^**^ | 5.2531 | 0.000001 |  |  |  |  |
| FC_4,5_ ^**^ | 4.9988 | 0.000004 |  |  |  |  |
| FC_3,4_ ^*^ | 2.3512 | 0.021 |  | FC_1,5_ ^*^ | -2.4956 | 0.0156 |
|  |  |  |  | FC_2,5_ ^*^ | -2.3042 | 0.0249 |
|  |  |  |  | FC_3,4_ ^*^ | -2.1295 | 0.0367 |
|  |  |  |  | FC_3,5_ ^*^ | -2.5324 | 0.0143 |

**Supplemental Table 4** Significantly different connections based on static EC (sEC) and variability of EC (vEC). The window width is set to 100 for constructing dynamic EC. * denotes that there is a difference by independent sample t test (p < 0.05).

| Static effective connectivity (sEC) | | |  | Variability of effective connectivity (vEC) | | |
| --- | --- | --- | --- | --- | --- | --- |
| Pair | T-statistic | P |  | Pair | T-statistic | P |
| EC_1,3_ ^*^ | -2.7915 | 0.0066 |  | EC_1,2_ ^*^ | -2.0120 | 0.0474 |
| EC_1,4_ ^*^ | -2.3518 | 0.0212 |  | EC_1,3_ ^*^ | -2.9616 | 0.0041 |
| EC_2,3_ ^*^ | -2.2402 | 0.0277 |  | EC_1,4_ ^*^ | -2.6675 | 0.0100 |
| EC_2,4_ ^*^ | -2.5870 | 0.0116 |  | EC_2,4_ ^*^ | -2.8850 | 0.0056 |


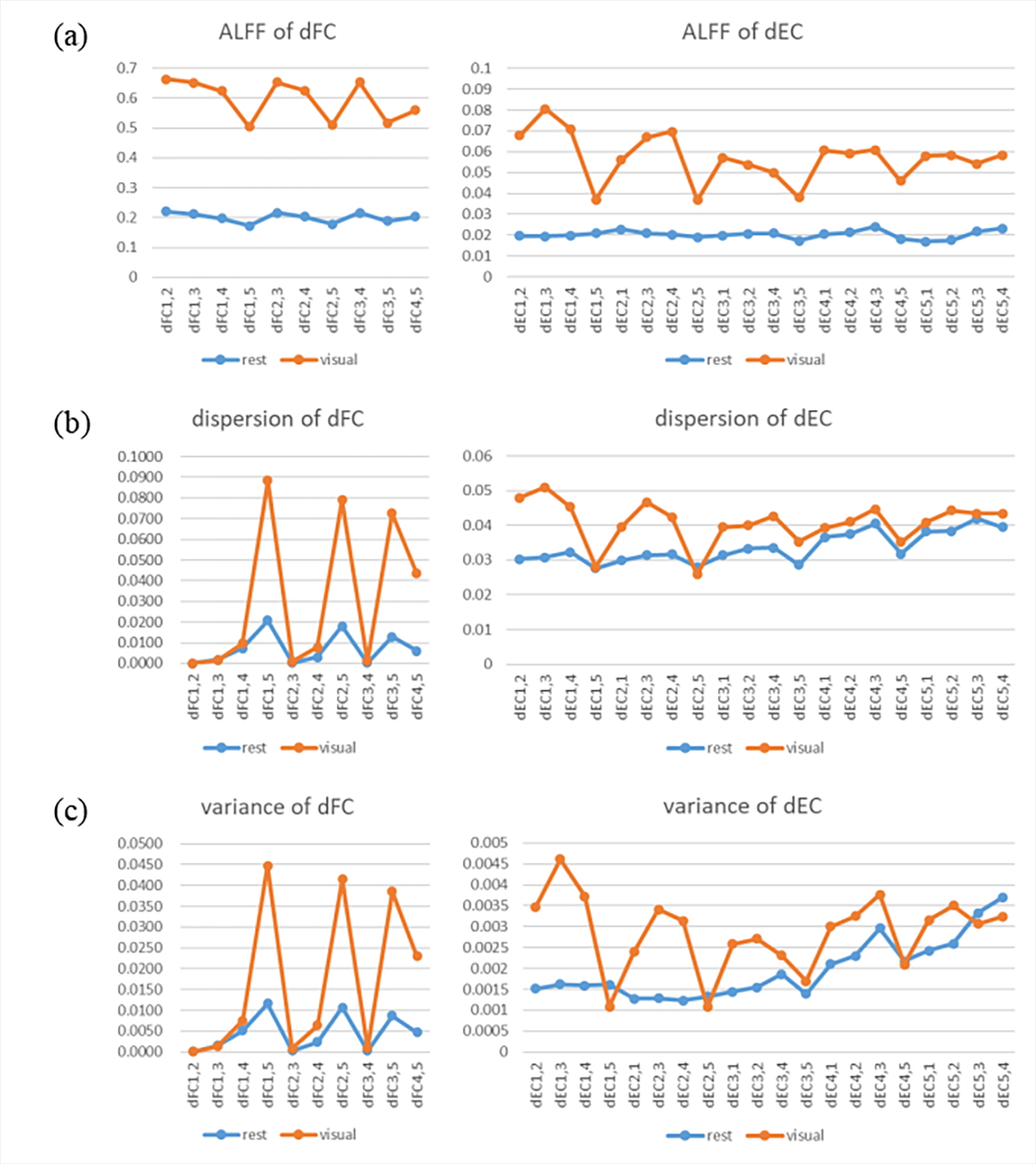


**Supplemental FIGURE 1** Results of the three dynamic measurement methods when the window width is set to 62. ALFF (a), dispersion (b), variance (c) of dynamic functional connectivity and effective connectivity are demonstrated respectively. dFCi,j is a vector which means time-varying functional connectivity (namely, changing trend) with elements calculated by Pearson correlation coefficient between ROIi and ROIj in all windows. dECi,j is a vector which means time-varying seffective connectivity (namely, changing trend) from ROIi to ROIj in all windows with elements calculated by Granger Causality method.


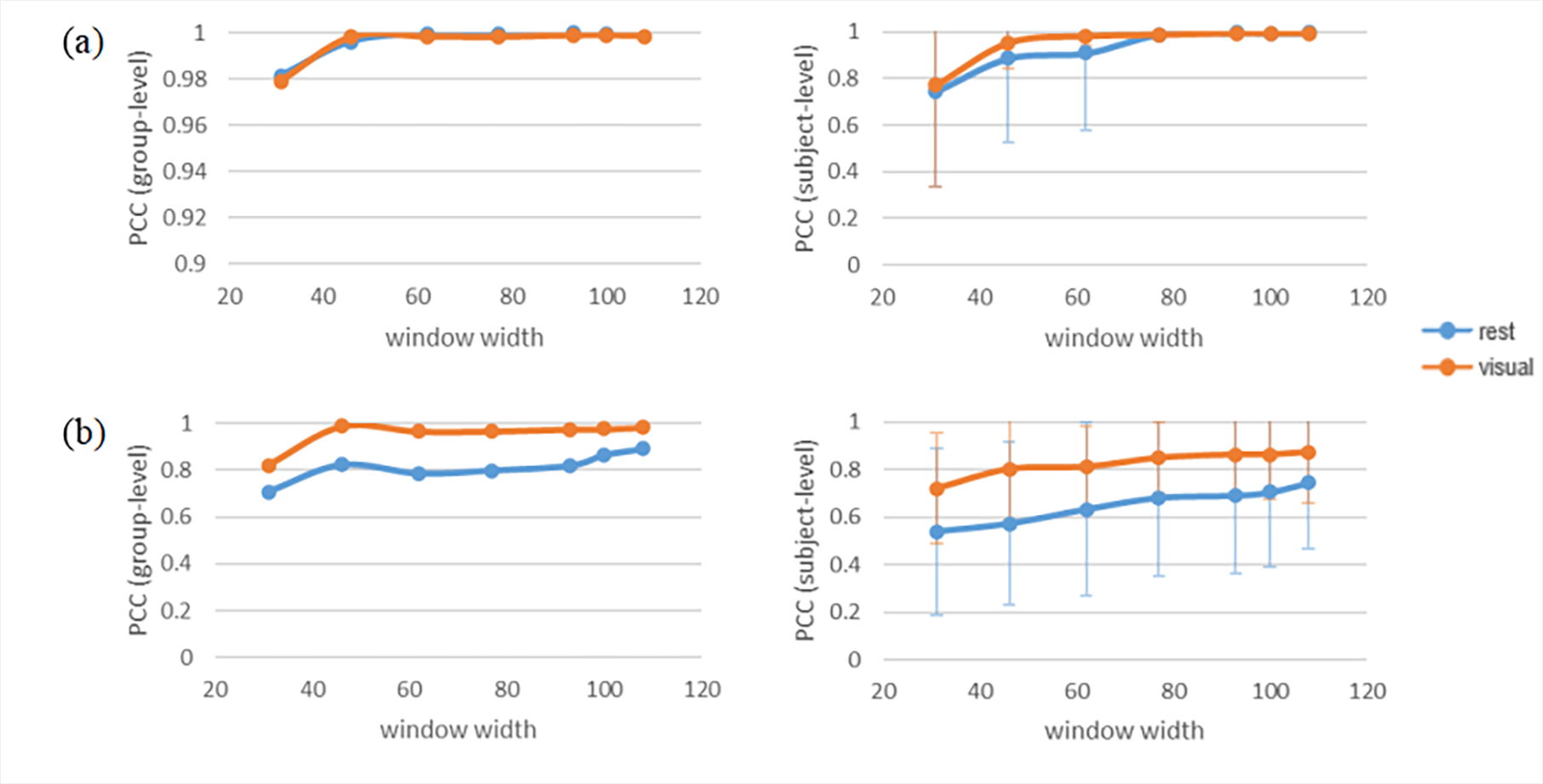


**Supplemental FIGURE 2** Pearson correlation coefficient between ALFF of dynamic FC and intensity of sFC (a), ALFF of dynamic EC and intensity of sEC (b) under different window width from group-level and subject-level, respectively.


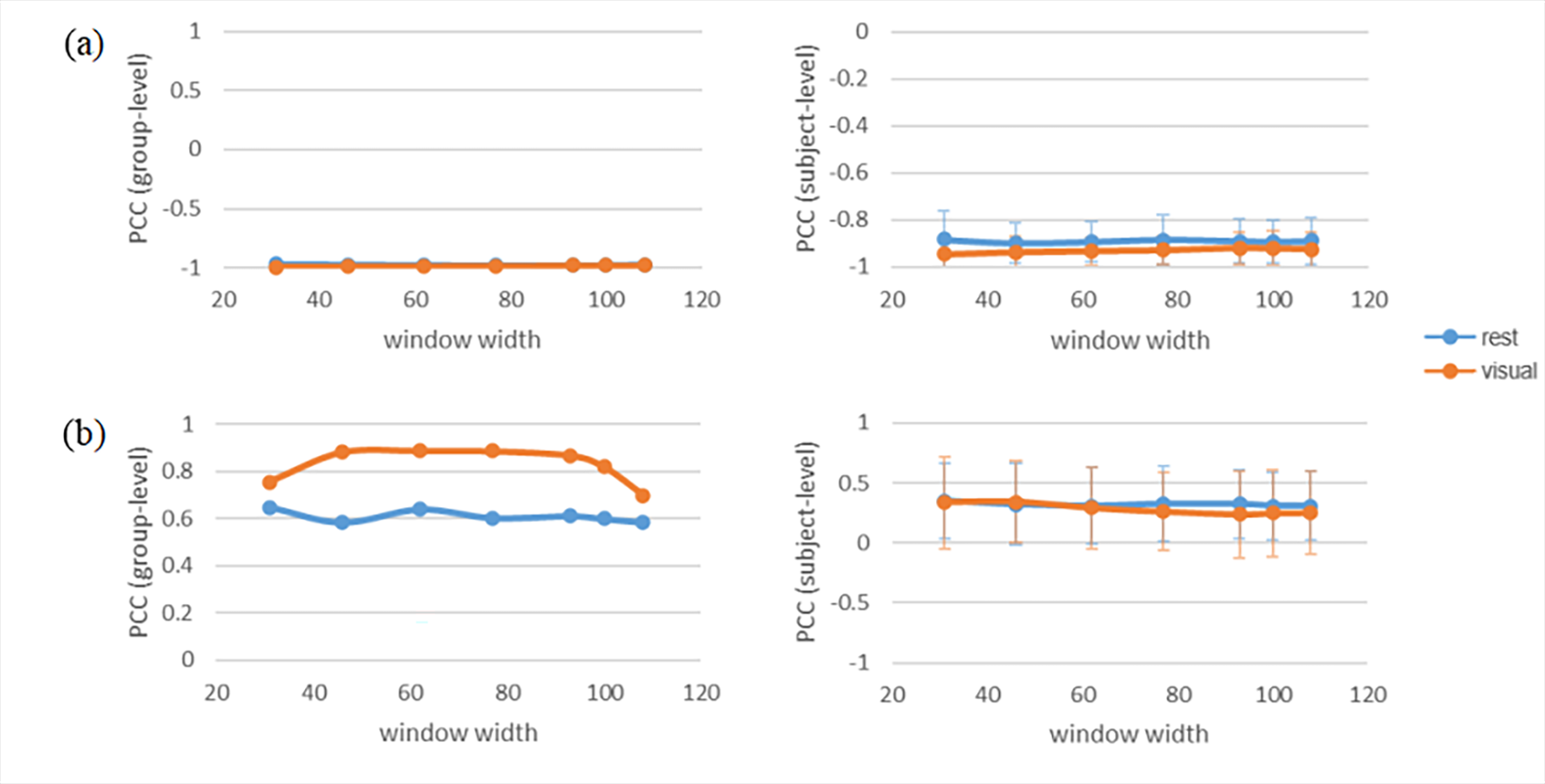


**Supplemental FIGURE 3** Pearson correlation coefficient between dispersion of dynamic FC and intensity of sFC (a), dispersion of dynamic EC and intensity of sEC (b) under different window width from group-level and subject-level, respectively.

To measure the impact of the sliding window size on relationship between static and dynamic connectivity, seven different window width were employed to calculate the Pearson correlation coefficient between intensity of static connectivity and three dynamic measurement methods (i.e. ALFF, dispersion and variance) (see **Supplemental Figure 2-3** and **Figure 9**). These results demonstrated that the influence of window size on PCC results was relatively minimal.
